# Robotic distal gastrectomy using a novel pre-emptive supra-pancreatic approach without duodenal transection in the dissection of D2 lymph nodes for gastric cancer

**DOI:** 10.3389/fonc.2024.1388626

**Published:** 2024-05-28

**Authors:** Jianming Xie, Jiabin Yang, Meixiao Wang, Yongfang Yin, Zhilong Yan

**Affiliations:** Department of Gastrointestinal Surgery, First Affiliated Hospital of Ningbo University, Ningbo, China

**Keywords:** robotic distal gastrectomy, pre-emptive supra-pancreatic approach, lymph node dissection, gastrointestinal surgery, gastric cancer

## Abstract

**Background:**

Robot-assisted surgery has shown remarkable progress as a minimally invasive procedure for gastric cancer. This study aimed to compare the pre-emptive suprapancreatic approach without duodenal transection and the conventional approach in terms of perioperative feasibility and short-term surgical outcomes.

**Methods:**

We retrospectively analyzed all patients who underwent robotic distal gastrectomy with D2 lymph node dissection using the da Vinci Xi robotic system between December 2021 and April 2023 and categorized them into two groups for comparison. Patients treated using the pre-emptive suprapancreatic approach (observation group) were compared with those who received the conventional approach (control group). Employing one-to-one propensity score matching, we evaluated the postoperative morbidity and short-term outcomes in these two distinct groups to assess the efficacy and safety of the novel surgical technique.

**Results:**

This study enrolled 131 patients: 70 in the observation group and 61 in the control group. After propensity score matching, the operative times were significantly longer in the control group than in the observation group (229.10 ± 33.96 vs. 174.84 ± 18.37, p <0.001). The mean blood loss was lower in the observation group than in the control group (25.20 ± 11.18 vs. 85.00 ± 38.78, p <0.001). Additionally, the observation group exhibited a higher number of retrieved lymph nodes, including suprapyloric, perigastric, and superior pancreatic lymph nodes (28.69 ± 5.48 vs. 19.21 ± 2.89, p <0.001; 4.98 ± 1.27 vs. 4.29 ± 1.21, p = 0.012; 10.52 ± 2.39 vs. 5.50 ± 1.62, p <0.001; 6.26 ± 2.64 vs. 5.00 ± 1.72, p = 0.029). Drain amylase levels in the observation group were significantly lower than those in the control group (30.08 ± 33.74 vs. 69.14 ± 66.81, p <0.001).

**Conclusion:**

This study revealed that using the pre-emptive suprapancreatic approach without duodenal transection in the dissection of D2 lymph nodes for gastric cancer is a safe and feasible procedure in terms of surgical outcomes.

## 1 Introduction

Gastric cancer (GC) is one of the most common malignant cancers of the digestive system and the second leading cause of cancer‐related deaths worldwide. Surgical resection is the primary curative treatment option (1). Robotic gastrectomy (RG) has become popular as a state-of-the-art, minimally invasive surgery for patients with GC. The da Vinci surgical system offers a high‐resolution three‐dimensional field of view and high degree of flexibility, which assists surgeons in overcoming the limitations of traditional methods (2). In 2003, Hashizume et al. (3) first used RG to treat GC. Subsequently, many authors reported their retrospective experience with RG for GC (4–6). Recently, several prospective studies and randomized controlled trials have provided evidence that RG for GC is technically safe and leads to more favorable short-term outcomes than laparoscopic gastrectomy for GC (7–9).

Deep and thorough lymph node dissection is key in robotic radical surgery for GC; however, challenges remain. The perigastric lymph nodes are mainly distributed around the blood vessels and centered on the pancreas. The course of perigastric blood vessels is complex, with many anatomical layers, and the adipose tissue of the lymph nodes is rich and deep (10). Moreover, because of the limitations of the Leonardo da Vinci robotic arm, it is impossible to achieve effective traction tension on the stomach and other large organs, and the lack of a tactile feedback system makes deep-seated complex lymphoid dissection challenging. Therefore, the selection of an appropriate surgical approach is key to the smooth implementation of robotic lymph node dissection for GC, achieving thorough perigastric lymph node dissection and greatly reducing complexities (11). Recognizing these challenges, our study aimed to explore alternative surgical methods to overcome these limitations. Innovatively, this study introduced a pre-emptive suprapancreatic approach without duodenal transection. Using the experience of our center, this study retrospectively analyzed 70 cases of radical gastrectomy using the da Vinci robotic pre-emptive suprapancreatic approach without duodenal transection with D2 lymph node dissection at the First Affiliated Hospital of Ningbo University between December 2021 and April 2023. This study aimed to compare this novel approach with the conventional method in terms of robotic distal gastrectomy outcomes.

## 2 Materials and methods

### 2.1 Patients

Data of 131 patients with GC who underwent robotic radical gastrectomy at the First Affiliated Hospital of Ningbo University between December 2021 and April 2023 were retrospectively analyzed. All patients met the following inclusion criteria (1): age >18 and <75 years (2), gastric adenocarcinoma diagnosed by pathological examination (3), clinical stage cT1-4aN0/+M0 assessed through preoperative evaluation, and (4) the possibility of R0 resection by distal subtotal gastrectomy and D2 lymph node dissection. The exclusion criteria were as follows (1): distant metastasis of GC or complications with other primary tumors (2); preoperative chemotherapy (3); American Society of Anesthesiology (ASA) score (physical status score) ≥4; and (4) incomplete case data. The cases were classified into two groups: the pre-emptive suprapancreatic approach without duodenal transection with D2 lymph node dissection (observation group) and conventional approach with D2 lymph node dissection (control group), in accordance with the fifth edition of the Japanese treatment guidelines for GC (12). The study enrolled 131 patients: 70 in the observation group and 61 in the control group. This study was approved by the Ethics Committee of the First Affiliated Hospital of Ningbo University. All the enrolled participants provided written informed consent.

### 2.2 Data collection

Data were collected from a prospectively maintained database at the First Affiliated Hospital of the Ningbo University. The data included demographic characteristics (sex, age, and body mass index [BMI]), preoperative data (ASA score, tumor status, and preoperative complications), intraoperative data (surgical approach, blood loss, and operation time), postoperative pathological diagnosis, and short-term outcomes (postoperative morbidity and mortality, length of postoperative hospital stay, drain amylase level, time to first postoperative fluid intake, time to first postoperative flatus, hospitalization expenses, and 1-year survival rate).

### 2.3 Operative procedure

In preparation for RG, the preoperative protocol emphasizes the need for detailed pathological and imaging assessments to tailor the treatment strategies. Through biopsy, gastric adenocarcinoma was confirmed and tumor histology and grading were identified, which is crucial for planning. Advanced imaging, including computed tomography and magnetic resonance imaging, allows for precise staging by revealing tumor size, location, and potential metastasis. This comprehensive evaluation aids in deciding the appropriateness of neoadjuvant therapy, aiming to reduce the tumor size for more effective resection. Decisions are made collaboratively to ensure that the treatment is optimized for each patient’s unique situation. This approach guarantees that all patients undergo rigorous evaluation, enhancing surgical success and long-term outcomes (13).

To minimize biases and ensure consistent results in our study, the same highly experienced surgical team performed all operations. Each surgeon had completed at least 100 laparoscopic- and robot-assisted radical distal gastrectomies, ensuring skill proficiency. This standardized experience aimed to limit the variability in operative times and outcomes due to surgeon expertise, focusing on our comparative analysis of the differences between surgical techniques.

All robotic distal gastrectomy procedures were performed using the da Vinci Xi surgical system with articulating robotic arms: a first arm for cadiere forceps, a second arm for fenestrated bipolar forceps, a third arm for a 30° rigid dual-channel endoscope, and a fourth arm for harmonic ACE-curved shears. An assistant port was placed at the left umbilical level.

**Figure 1:**
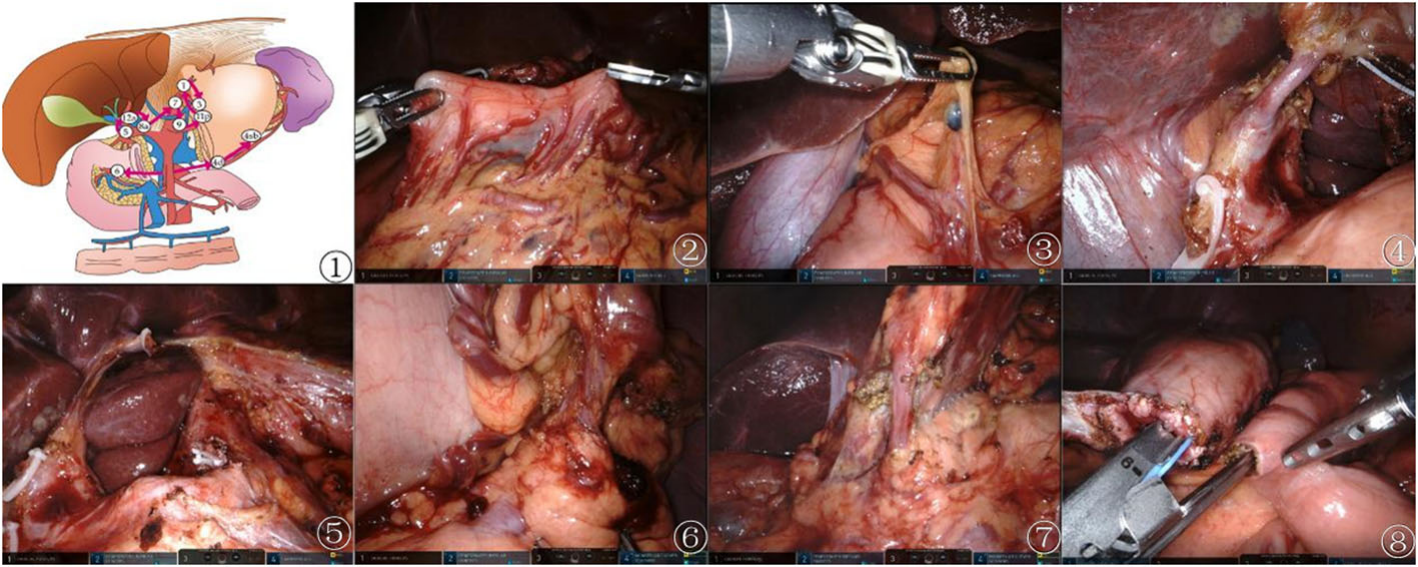
Preemptive supra-pancreatic approach without duodenal transection. ① Schematic diagram of the novel approach. ② Tumor localization. ③ Dissection of suprapyloric lymph nodes. ④\⑤ Dissection of superior pancreatic lymph nodes. ⑥ Dissection of NO.4sb lymph nodes. ⑦ Dissection of Subpyloric lymph nodes. ⑧ Billroth II gastrojejunal anastomosis.

In the observation group, D2 lymph node dissection followed a specific sequence of lymph node stations (5, 12a, etc.), employing harmonic ACE curved shears and other techniques to meticulously expose and dissect targeted lymph nodes (including stations 5, 12a, 8a, 9, 7, 11p, 3, 1, 6, 4sb, and 4d), with minimal trauma. The techniques involved included retraction of the stomach, exposure of critical arteries, and dissection along predetermined lymphatic stations to ensure comprehensive removal. The dissection of the control group adhered to traditional guidelines. This detailed approach aims to maximize cancer clearance while minimizing damage (13). The digestive tract was reconstructed through an intracorporeal antecolic Billroth II gastrojejunal anastomosis (**Figure 1**).

### 2.4 Follow-up monitoring after surgery

Patients with pathological stage II or III cancer received adjuvant chemotherapy with S-1 for 1 year (13). Data on prognosis were collected every 3 months.

### 2.5 Statistical analysis

All statistical analyses were performed using SPSS version 25.0 (SPSS Inc., Chicago, IL, USA). Independent continuous variables were expressed as mean ± standard deviation and compared using Student’s t-test or Mann–Whitney U test. Categorical variables were compared using the chi-squared test or Fisher’s exact test. Statistical significance was set at p-value <0.05. Propensity score matching (PSM) was conducted to overcome possible patient selection bias and remove confounding factors. The variables included age, sex, BMI, ASA score, preoperative complications, tumor size, pT stage, pN stage, pTNM stage, and histological type. Patients in the observation and control groups were matched in a 1:1 ratio using the nearest propensity score on the logit scale. The caliper value was set at 0.02. Overall survival (OS) was defined as days from gastrectomy to death; it was assessed through Kaplan–Meier analysis and compared using the log-rank test.

## 3 Results

### 3.1 Baseline characteristics

Patient characteristics are summarized in **Table 1**. Before PSM, the control group contained more male patients than did the observation group (61.3% vs. 45.9%, p = 0.010). The mean age of the observation group was significantly younger than that of the control group (59.2 ± 12.52 vs. 64.68 ± 9.64, p = 0.005). The mean tumor size of the observation group was significantly larger than that of the control group (3.98 ± 2.11 vs. 3.32 ± 2.08, p = 0.047).

**Table 1:** Demographics and clinical characteristics between the observation groups and control groups before and after matching.

Variables	Entire cohort			Propensity score-matched cohort		
	Control group (n = 61)	Observation group (n = 70)	p	Observation Group (n = 45)	Control Group (n = 45)	P
Age, years	64.68 ± 9.64	59.2 ± 12.52	0.005	59.78 ± 8.72	61.23 ± 8.23	0.693
Sex, N0. (%)			0.010			0.103
Male	38 (61.3)	28 (45.9)		27 (60.0)	24 (53.3)	
female	23 (38.7)	42 (54.1)		18 (40.0)	21 (46.7)	
BMI, kg/m^2^	22.82 ± 2.38	23.10 ± 3.51	0.606	22.45 ± 1.89	23.05 ± 2.87	0.701
ASA score, N0. (%)			0.258			0.603
I	54 (88.5)	60 (85.7)		40 (88.9)	39 (86.7)	
II	6 (9.8)	7 (10)		4 (8.9)	5 (11.1)	
≥III	1 (1.7)	3 (4.3)		1 (2.2)	1 (2.2)	
Comorbidity, N0. (%)			0.738			0.648
no comorbidity	27 (44.2)	25 (35.7)		23 (51.1)	15 (33.3)	
Cardiovascular	21 (34.4)	25 (35.7)		13 (28.9)	18 (40)	
Diabetes type 2	7 (11.5)	11 (15.7)		6 (13.3)	7 (15.6)	
Viral hepatitis	2 (3.3)	5 (7.1)		1 (2.2)	3 (6.7)	
Cerebrovascular	4 (6.6)	4 (5.7)		2 (4.5)	2 (4.4)	
Size, (cm)	3.32 ± 2.08	3.98 ± 2.11	0.047	3.15 ± 1.54	3.34 ± 2.50	0.08
pT stage, No. (%)			0.279			0.295
T1	29 (47.5)	22 (31.4)		22 (48.9)	15 (33.3)	
T2	9 (14.8)	11 (18.0)		6 (13.3)	9 (20.0)	
T3	12 (19.7)	20 (28.6)		9 (20.0)	15 (33.3)	
T4	11 (18.0)	17 (12.0)		8 (17.8)	6(13.3)	
pN stage, No. (%)			0.557			0.495
N0	35 (57.3)	38 (54.3)		27 (60.0)	30 (66.7)	
N1	9 (14.8)	15 (21.4)		6 (13.3)	10 (22.2)	
N2	7 (11.5)	10 (14.3)		4 (8.9)	9 (20.0)	
N3	10 (16.4)	7 (10.0)		8 (17.8)	6 (13.3)	
P stage			0.815			0.346
I	33 (54.1)	36 (51.4)		25 (55.6)	30 (66.7)	
II	8 (13.1)	12 (17.2)		6 (13.3)	8 (17.8)	
III	20 (32.8)	22 (31.4)		14 (31.1)	7 (15.5)	
Histological type, No. (%)						
Well/moderate	7 (11.5)	10 (14.3)	0.633	4 (8.9)	5 (11.1)	0.500
Poor/undifferentiated	54 (88.5)	60 (85.7)		41 (91.1)	40 (88.9)	

ASA, American Society of Anesthesiologists; BMI, body mass index. The χ2 test or Fisher’s exact test was used for between-group comparison of Sex, ASA score, Comorbidity, Histological type, pT, pN, and pStage. The student’s t-test or Mann–Whitney U test was applied for between-group comparison of BMI, tumor size.

No significant differences were observed between the groups in terms of BMI, ASA score, comorbidity, pathologic TNM stage, or histological type. After PSM, 45 matched pairs were selected, and the baseline characteristics were well-balanced between the observation and control groups.

### 3.2 Short−term surgical outcomes

Surgical results, postoperative recovery, and postoperative complications after PSM are summarized in **Table 2**. Operative times were significantly longer in the control group than in the observation group (229.10 ± 33.96 vs. 174.84 ± 18.37, p <0.001). Mean blood loss was lower in the observation group than in the control group (25.20 ± 11.18 vs. 85.00 ± 38.78, p <0.001).

**Table 2:** Surgical results, postoperative recovery, and postoperative complications after matching.

Variables	Observation group (n = 45)	Control group (n = 45)	P-value
Operation time, (min)	174.84 ± 18.37	229.10 ± 33.96	<0.001
Blood loss, (ml)	25.20 ± 11.18	85.00 ± 38.78	<0.001
Retrieved lymph nodes	28.69 ± 5.48	19.21 ± 2.89	<0.001
Suprapyloric lymph nodespy (5, 12a)	4.98 ± 1.27	4.29 ± 1.21	0.012
superior pancreatic lymph nodes (7, 8, 9, 11p)	10.52 ± 2.39	5.50 ± 1.62	<0.001
Subpyloric lymph nodes (6)	4.67 ± 1.33	4.50 ± 1.07	0.574
Perigastric lymph nodes (1, 3, 4)	6.26 ± 2.64	5.00 ± 1.72	0.029
Number of metastatic LNs	2.66 ± 4.53	3.89 ± 4.27	0.060
Postoperative recovery	2.66 ± 4.5	3.89 ± 4.27	0.060
Time to first flatus	25.84 ± 10.75	31.29 ± 14.74	0.065
Time to liquid intake	48.98 ± 12.06	33.54 ± 15.72	0.063
Postoperative hospital stay (days)	9.08 ± 3.09	10.21 ± 4.47	0.059
Postoperative complications (CD classification) No. (%)			0.500
Overall complications, grade I/II	6 (13.33)	5 (11.11)	
Overall complications, grade≥IIIa	1 (2.22)	2 (4.44)	
Mortality	0 (0.00)	0 (0.00)	
Medical cost (RMB)	94,382.596 ± 15,383.67	97,231.69 ± 16,341.29	0.067
D.AMY(U/L)	30.08 ± 33.74	69.14 ± 66.81	<0.001

CD classification, Clavien-Dindo’s classification of surgical complication; D.AMY, drain amylase concentration in the drainage fluid at postoperative day 3. The Student’s t-test or the Mann–Whitney U test was applied for between-group comparison of operative time, blood loss, number of retrieved LNs, time to first flatus, time to liquid intake, postoperative hospital stay and D.AMY; The χ^2^ test as used for between-group comparison of Postoperative complications.

The observation group had more retrieved lymph nodes, suprapyloric lymph nodes (5, 12a), superior pancreatic lymph nodes (7, 8, 9, 11p) and perigastric lymph nodes (1, 3, 4) than the control group (28.69 ± 5.48 vs. 19.21 ± 2.89, p <0.001; 4.98 ± 1.27 vs. 4.29 ± 1.21, p = 0.012; 10.52 ± 2.39 vs. 5.50 ± 1.62, p <0.001; 6.26 ± 2.64 vs. 5.00 ± 1.72, p = 0.029). Drain amylase levels in the observation group were significantly lower than those in the control group (30.08 ± 33.74 vs. 69.14 ± 66.81, p <0.001). The differences between the groups in terms of retrieved subpyloric lymph nodes (6), number of metastatic lymph nodes, postoperative recovery, time to first flatus, time to liquid intake, postoperative hospital stay, postoperative complications, and medical cost were not significant. No mortality was noted within 90 days of the surgery.

### 3.3 Survival analysis

**Figure 2:**
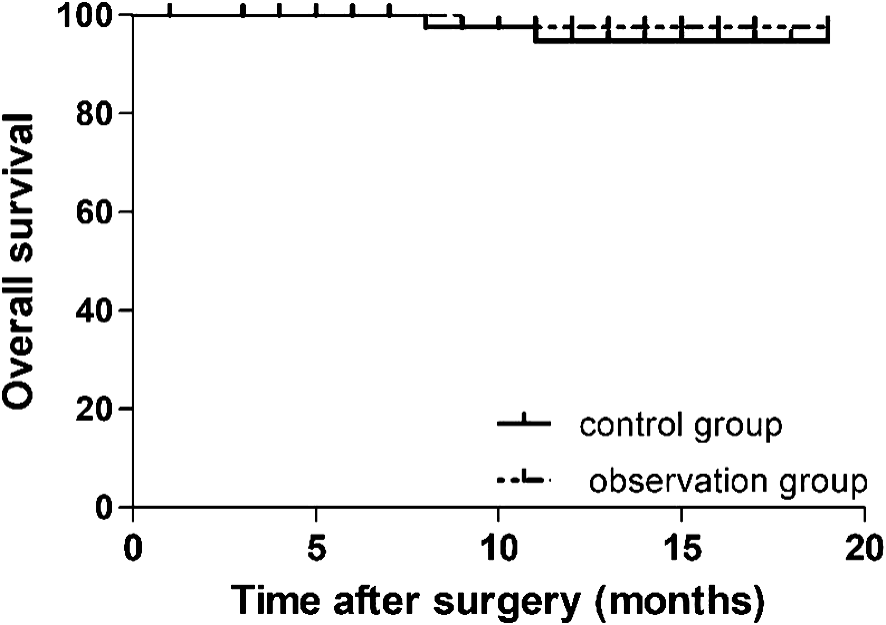
Cumulative survival curves for patients undergoing preemptive suprapancreatic approach without duodenal transection and the conventional approach after propensity score matching. The 1-year overall survival rate did not differ significantly between the observation and control groups (97.8% vs.95.6%, respectively; log-rank *p* = 0.543).

The data of 90 patients between December 2021 and April 2023 were considered for the survival analysis. The follow-up period was 1–19 months and the median follow-up time was 12 months. The 1-year survival rates in the observation and control groups were 97.8% and 95.6%, respectively. There was no statistically significant difference in the OS time between the two groups (log-rank p = 0.543) (**Figure 2**).

## 4 Discussion

This single-center PSM study revealed encouraging results for the novel pre-emptive suprapancreatic approach without duodenal transection in RG by comparing the short-term surgical outcomes of this novel approach with those of conventional RG. The results demonstrated that the pre-emptive suprapancreatic approach significantly reduced operative time and intraoperative blood loss compared with conventional RG. Thus, the novel preemptive suprapancreatic approach to RG may be an effective alternative for the treatment of GC.

Accurate lymph node dissection is the focus and greatest difficulty in RG. The order of lymph node dissection in the traditional approach is frequently from the greater curvature of the stomach to the lesser curvature and from the caudal side to the head side (14, 15). Although the da Vinci surgical system has a larger three-dimensional surgical field of vision and a flexible robotic arm, it has only one robotic arm to assist in the exposure and lacks force feedback. Therefore, it is impossible to achieve effective traction tension on the stomach and other large organs, resulting in challenges for deep-seated complex lymphoid dissection (16, 17). Therefore, we propose a new surgical approach, the preemptive suprapancreatic approach, without duodenal transection. This surgical approach changes the order of lymphadenectomy, reducing visual field transformation and tissue flipping to avoid tissue traction injury. In addition, it reduces the traction on and flipping of the stomach wall, which is more aligned with the principle of tumor-free outcomes and increases surgery coherence. This approach is beneficial for maintaining an open field of vision in the superior pancreatic area and reducing the difficulty of lymphadenectomy. In this study, the observation and control groups yielded 28.69 ± 5.48 and 19.21 ± 2.89 retrieved lymph nodes, respectively, which exceeded the recommended number of retrieved lymph nodes (18, 19). Additionally, the total number of retrieved lymph nodes in the observation group was significantly higher than that in the control group. Further stratified analysis revealed that the observation group had a significantly higher number of lymph nodes retrieved from the superior pancreatic lymph nodes (7, 8, 9, 11p), perigastric lymph nodes (1, 3, 4), and suprapyloric lymph nodes (5, 12a) than the control group. Therefore, the preemptive suprapancreatic approach may provide clinicians with more accurate staging information and reduce postoperative locoregional recurrence rates. Dissecting more lymph nodes may lead to increased operative time and bleeding; hence, dissecting lymph nodes without increasing the operative time or causing excessive blood loss is crucial (20, 21). This study demonstrated that the differences in the mean operative time and mean amount of blood loss between the groups were significant.

The presence of postoperative complications is important when assessing the quality and safety of operations (22, 23). Dissection of interstitial tissues around the pancreas forms the cornerstone of gastric cancer surgery (24), inevitably leading to potential pancreatic damage and severe complications. Studies have indicated that the technical advantages of robotic surgical systems facilitate the dissection of suprapancreatic lymph nodes, potentially reducing the likelihood of pancreatic injury (25). In our study, we found that the incidence of postoperative complications was comparable between the observation and the control groups. Nonetheless, the observation group exhibited a tendency towards lower levels of drain amylase, suggesting that robotic gastrectomy employing a novel preemptive approach to suprapancreatic dissection may effectively minimize pancreatic damage.

To successfully perform the preemptive suprapancreatic approach and shorten the learning curve of the new technique, the following points should be noted (1). The suprapyloric area should be a priority for dissection. There was a relatively fluid line between lymph node stations 8 and 5. Cutting off the right gastric vessels at the root followed by cutting the suprapyloric vessels can significantly increase the degree of freedom of the gastric antrum (2). The hepatogastric ligament should be fully opened, and the lesser omentum should be incised perpendicular to the stomach wall at approximately the same angle as the stomach until the lesser curvature of the stomach is reached, increasing the degree of freedom of the stomach and exposing the root area of the splenic artery (3). Lymph node dissection should be performed according to the region and in line with conventions to minimize visual field transformation and tissue flipping and increase the fluency of the operation.

Addressing the disparities in surgical outcomes across different centers, which are directly related to the capabilities and resources available, is crucial. The advanced RG technique highlighted in this study underscores the significant role of specialized technical expertise, the necessity for cutting-edge equipment, and the value of a well-coordinated multidisciplinary team. These factors are not uniformly present across all medical institutions, which explains the variation observed in the success rates. Highly skilled surgeons trained specifically in robotic surgery and the availability of the latest robotic systems are prerequisites for such complex procedures. Moreover, seamless integration of care through a multidisciplinary team approach enhances patient outcomes from preoperative assessment to postoperative recovery. Therefore, not all centers can readily implement these advanced techniques without substantial investment in human and technological resources. This understanding should guide institutions in evaluating their readiness to adopt such procedures, with an emphasis on the need for specialized training, equipment acquisition, and fostering a collaborative environment. Our findings suggest that adherence to these principles is essential for achieving optimal surgical results and should therefore be considered a benchmark for centers looking to advance their surgical capabilities.

This study has several limitations. This was a retrospective study conducted at a single center and a randomized controlled trial was not performed. Moreover, the long-term oncological outcomes have not yet been investigated.

### 4.1 Conclusions

In conclusion, this study revealed that using the preemptive suprapancreatic approach without duodenal transection in the dissection of D2 lymph nodes for GC is safe and feasible in terms of surgical outcomes.

## Data availability statement

The original contributions presented in the study are included in the article/supplementary material. Further inquiries can be directed to the corresponding author.

## Ethics statement

The studies involving humans were approved by the Ethics Committee of the First Affiliated Hospital of Ningbo University. The studies were conducted in accordance with the local legislation and institutional requirements. The participants provided their written informed consent to participate in this study.

## Author contributions

JX: Conceptualization, Data curation, Formal analysis, Investigation, Project administration, Resources, Writing – original draft. JY: Data curation, Investigation, Methodology, Resources, Software, Validation, Writing – original draft. MW: Data curation, Formal analysis, Investigation, Methodology, Writing – original draft. YY: Data curation, Formal analysis, Investigation, Resources, Validation, Writing – original draft. ZY: Conceptualization, Data curation, Formal analysis, Investigation, Methodology, Project administration, Resources, Software, Writing – review & editing.
